# Delayed Post-hypoxic Leukoencephalopathy: A Case Series and Review of the Literature

**DOI:** 10.7759/cureus.2481

**Published:** 2018-04-15

**Authors:** Nakul Katyal, Naureen Narula, Pravin George, Premkumar Nattanamai, Christopher R Newey, Jonathan M Beary

**Affiliations:** 1 Department of Neurology, University of Missouri, Columbia, USA; 2 Internal Medicine, Staten Island University Hospital, staten island, USA; 3 Neurology, Cleveland Clinic Ohio; 4 Neurobehavioral Sciences, A. T. Still University

**Keywords:** post hypoxic leukoencephalopathy

## Abstract

Delayed post-hypoxic leukoencephalopathy (DPHL) is a unique clinical entity that presents with cognitive impairment days to weeks after an episode of acute hypoxic brain injury. Frequently hypoxia is unrecognized as a mechanism for clinical decline and extensive workup ensues. We present two cases of DPHL highlighting the neuroimaging findings. In both patients, a cerebral hypoxic event was followed by a recovery phase with subsequent delayed clinical decline. Patient 1 suffered hypoxia from drug-induced respiratory depression and lack of post-operative positive airway pressure (PAP) support. Her neurological exam on follow-up revealed progressive cognitive decline. Magnetic resonance imaging (MRI) brain showed bilateral white matter changes involving the centrum semiovale. Patient 2 developed a generalized tonic-clonic seizure during an endobronchial biopsy procedure and was found to have multiple air emboli on computed tomography (CT) head scan. She was initially in a drug-induced coma for her seizures. Electroencephalography (EEG) on day 14 of admission showed changes consistent with diffuse encephalopathy. MRI brain showed bilateral white matter changes particularly at the watershed zones and in the centrum semiovale. DPHL is a rare and under-recognized clinical entity that requires clinical suspicion and detailed evaluation for diagnosis. Neuroimaging studies can provide prognostic information regarding the extent of neurological injury.

## Introduction

Delayed post-hypoxic leukoencephalopathy (DPHL) is a clinical syndrome of delayed cognitive decline in a patient with an anticedent hypoxic event. Patients typically present one and four weeks after a hypoxic event for evaluation of encephalopathy. Although multiple possible mechanisms have been proposed to explain its delayed manifestation, the exact mechanism of DPHL remains elusive [1]. The earliest reported case was related to carbon monoxide (CO) poisoning [2-4]. Plum et al. reported several cases of DPHL related to surgical anesthesia complications, cardiac arrest, or CO poisoning [5]. Multiple other presentations have been reported in settings of strangulation [6], hemorrhagic shock [7], and overdoses of opiates and/or benzodiazepines [8,9]. We present two DPHL patients with distinct etiologies. We describe these cases and review the literature on clinical and neuroimaging presentation of DPHL.

## Case presentation

Case 1

**Figure 1:**
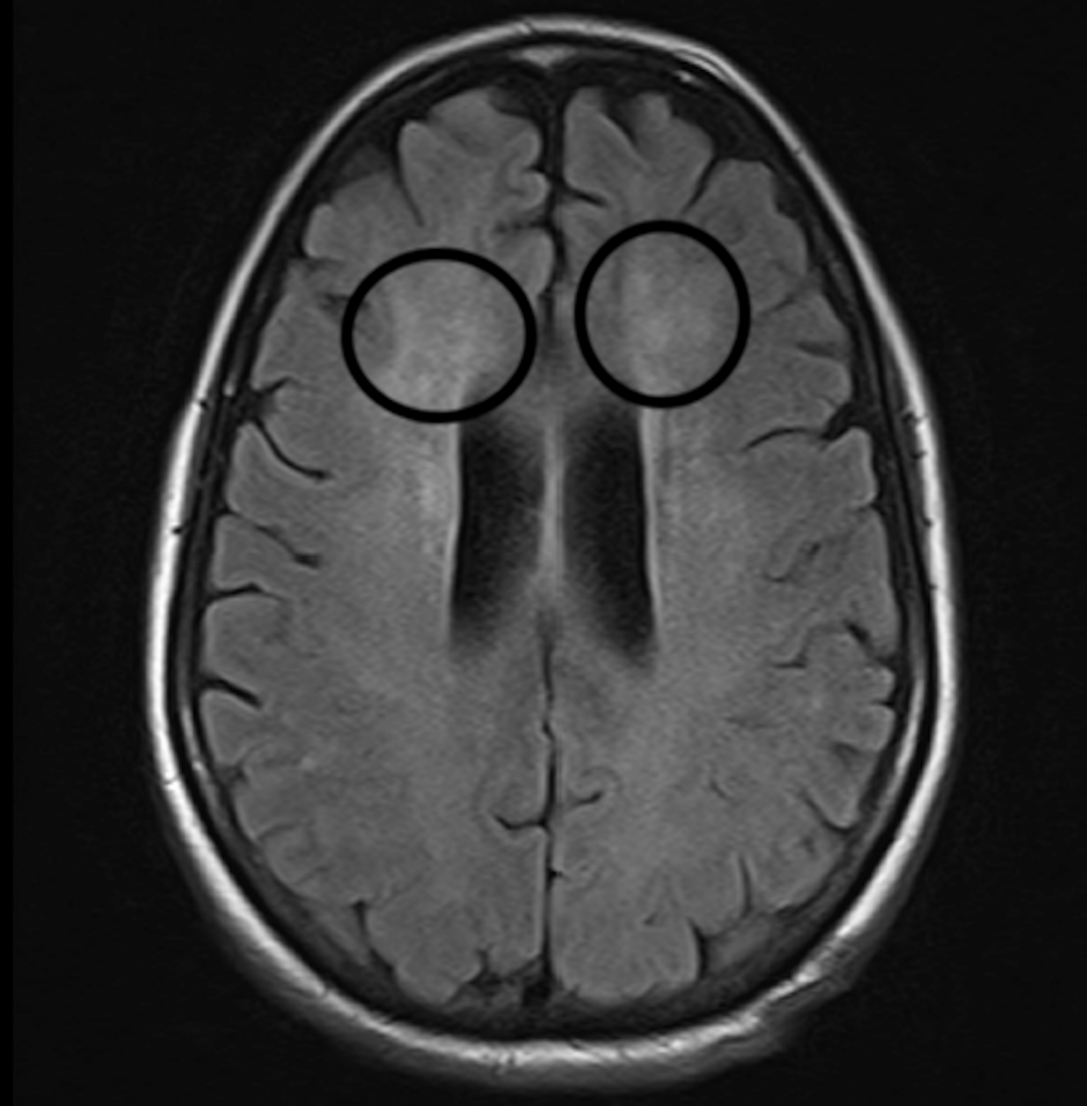
Magnetic resonance imaging (MRI). MRI brain showing nonspecific white matter changes supratentorially (circles).

A 59-year-old left-handed female with a history of hypertension, steatohepatitis, hypothyroidism, and obstructive sleep apnea (OSA) was brought to the emergency department (ED) with progressive altered mental status, abulia, and inability to care for herself. The family reported inadequate dietary intake and increasing forgetfulness in the last week. Her history was significant for laparoscopic Roux-en-Y gastric bypass bariatric surgery one month prior. The post-operative course was uneventful, and she was discharged home with normal mental status on nightly continuous positive airway pressure (CPAP). Three days later she was brought to the ED in a lethargic state after falling out of bed in the setting of CPAP noncompliance. She was admitted to the surgical intensive care unit for acute hypoxic respiratory failure and was intubated. Computed tomography (CT) chest only showed small bilateral pleural effusions. She was eventually extubated and placed on a regimen of CPAP when asleep and transferred to the regular nursing floor. Despite adequate oxygenation, the patient remained arousable but disoriented with decreased attention span. Cranial nerve, motor and sensory examinations were normal. Magnetic resonance imaging (MRI) brain showed nonspecific white matter disease of the centrum semiovale (Figure 1).

Lumbar puncture revealed an elevated myelin basic protein. Her vitamin D-25 and methylmalonic acid levels were low. Her thyroid function workup was consistent with hypothyroidism. The rest of her metabolic workup was unremarkable. She was eventually discharged to a skilled nursing facility (SNF) with neurology follow-up.

Case 2

**Figure 2:**
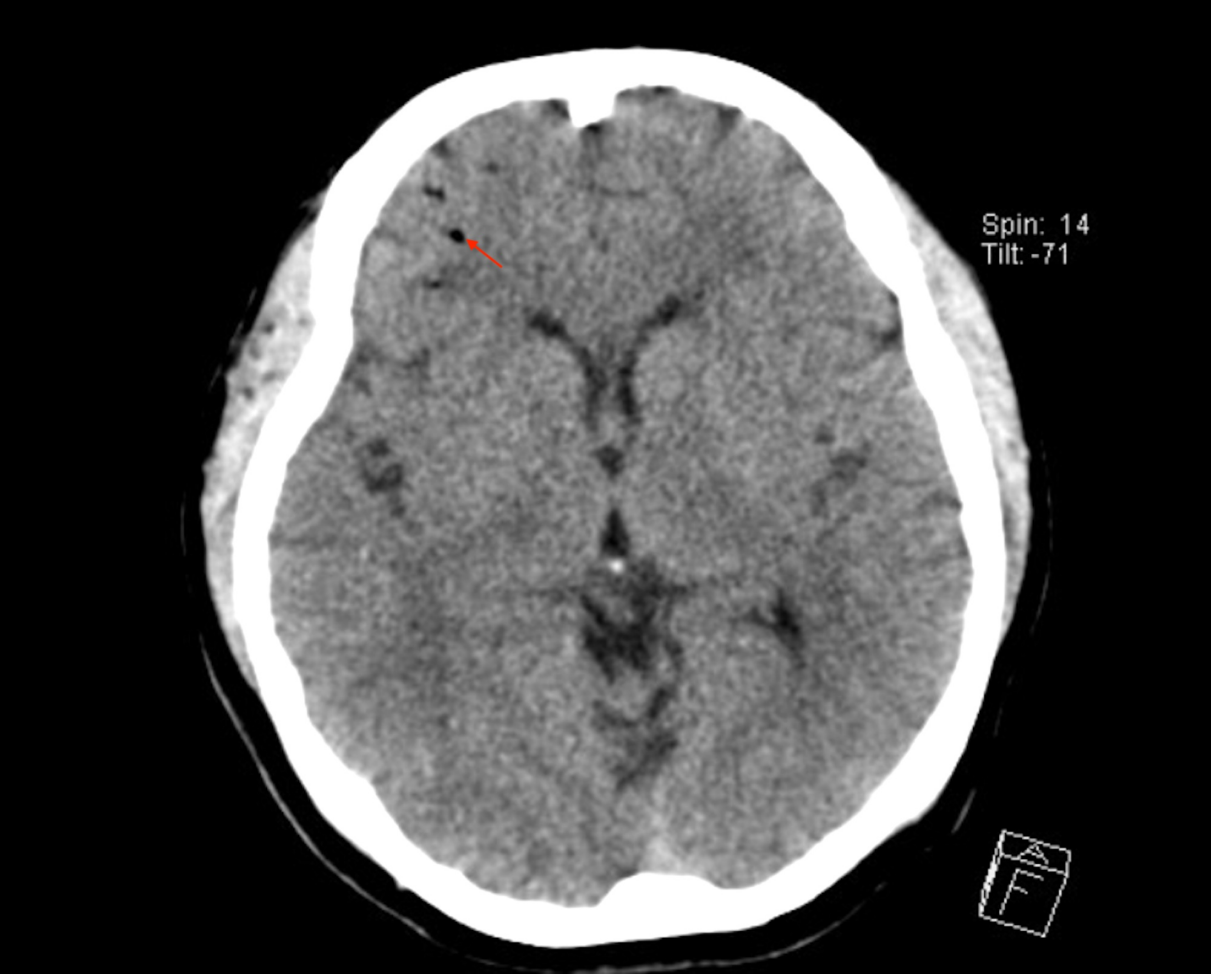
Computed tomography (CT) head. CT head showing multiple air emboli (arrow).

A 71-year-old female with a history of in situ ovarian adenocarcinoma status post appendectomy and right-sided hemicolectomy developed generalized tonic-clonic seizure activity. An initial seizure was noted while undergoing an endobronchial biopsy procedure for evaluation of a perihilar mass. Pathology was consistent with a benign reactive lymph node. During the procedure, she developed mottled discoloration of her skin, spreading from her abdomen to both shoulders. Bag ventilation was started, and she was transferred to the surgical intensive care unit (ICU) and intubated. CT head revealed multiple air emboli (Figure 2).

**Figure 3:**
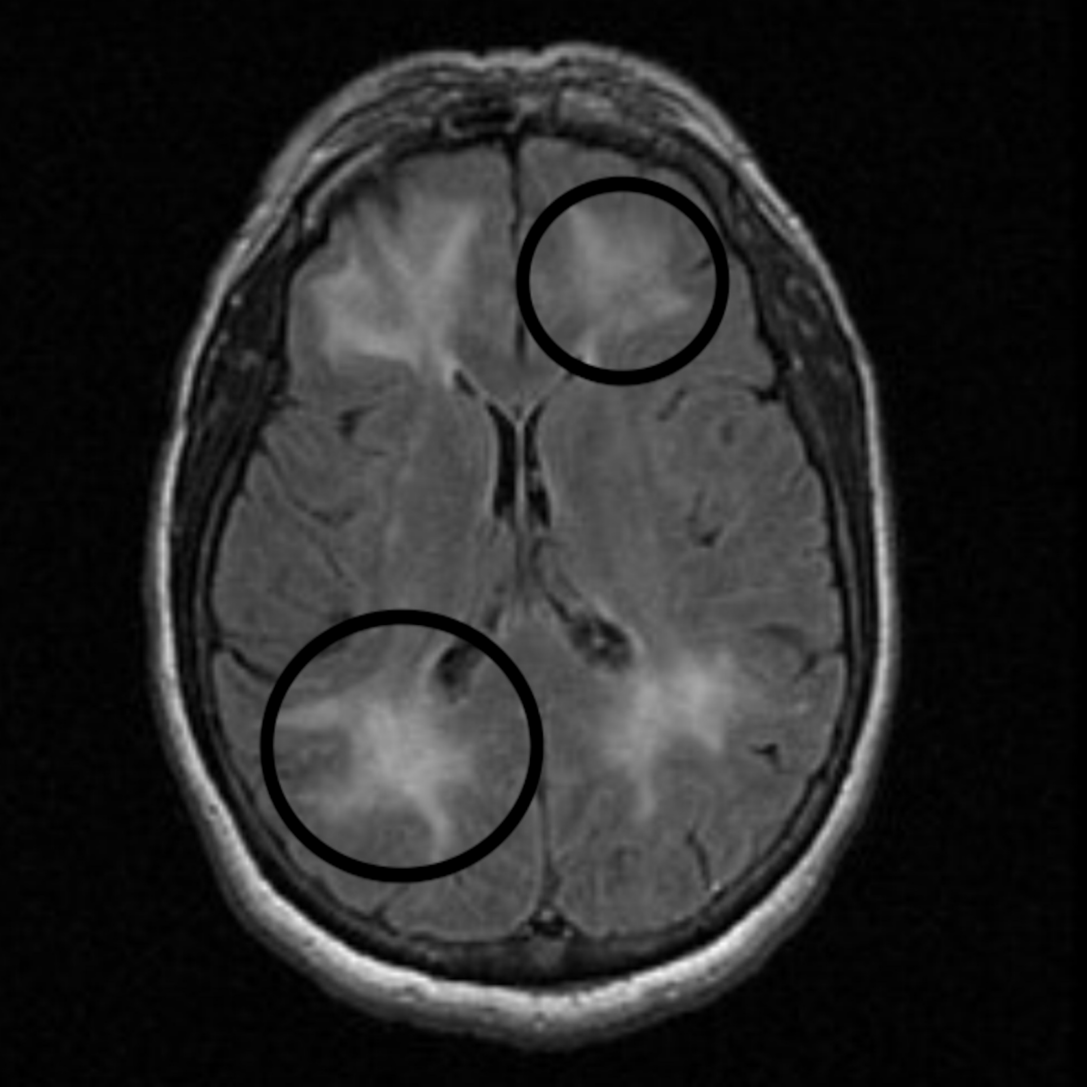
Magnetic resonance imaging (MRI). MRI brain scan showing diffuse white matter changes (circles).

Continuous electroencephalography (CEEG) monitoring revealed frequent periodic lateralized epileptiform discharges. Her anti-epileptic medication was rapidly escalated to pentobarbital in addition to levetiracetam, lacosamide, and phenytoin. Her Glasgow Coma Scale was 3 (E:1;V:1;M:1). Neurological examination was significant for sluggish but reactive bilateral pupils and areflexic quadriplegia. She was transferred to the neurological ICU at that time. Her neurological examination remained same thereafter. On day 14 of her admission, EEG showed changes consistent with bilateral cortical dysfunction in bifrontal regions indicating severe diffuse encephalopathy. No seizure activity was noted on EEG. Cerebrospinal fluid (CSF) analysis revealed an elevated myelin basic protein but with undetected white blood cells (WBCs), red blood cells (RBCs), and negative cytology. MRI brain scan the following day showed progressive diffuse white matter changes in a watershed distribution and centrum semiovale (Figure 3).

She was weaned from her antiepileptic regimen to levetiracetam monotherapy. She slowly improved, but required tracheostomy and percutaneous gastrostomy tube. She was eventually transferred to an acute rehabilitation facility.

## Discussion

Our cases highlight two distinct presentations of delayed onset neurological dysfunction in patients with preceding hypoxia. Neurological deficits appeared following an initial phase of recovery in both patients. The clinical and MRI findings suggested DPHL. DPHL is an under-recognized clinical entity where patients classically develop cognitive decline after initial recovery from an acute hypoxic episode [1]. Diagnosis is usually confirmed after excluding other potential causes of acute altered mental status, such as the history of CO poisoning, narcotic overdose, or other global cerebral hypoxic event. Neuroimaging reveals characteristic diffuse hemispheric demyelination sparing the cerebellum and the brainstem tracts [2]. Plum et al. reported several cases of DPHL related to surgical anesthesia complications, cardiac arrest, or CO poisoning [5]. Multiple other presentations have been reported in settings of strangulation [6], hemorrhagic shock [7], and overdoses of opiates and/or benzodiazepines [8,9]. The lucid interval usually lasts between seven and 21 days but ranges from two to 40 days.

Multiple possible mechanisms are described in the literature, however, the exact mechanism explaining DPHL remains unclear. Cell death after cerebral ischemia was initially considered to be necrotic in nature, but recent studies have revealed that in cases of cerebral hypoxia many neurons in the ischemic penumbra undergo apoptosis. Cerebral hypoxia generally triggers two pathways of apoptosis: the intrinsic pathway, originating from mitochondrial release of cytochrome C and associated stimulation of caspase-3; and the extrinsic pathway, originating from the activation of cell surface death receptors, resulting in the stimulation of caspase-8 [10]. Although morphologically distinct, both necrosis and apoptosis are part of the continuum of cell death with similar operative mechanisms. Necrosis is a rapidly occurring form of cell death resulting from imbalances in ionic homeostasis following hypoxic injury. Apoptosis is generally a delayed form of cell death resulting from activation of intrinsic and extrinsic pathways. Following hypoxia-ischemia, excitatory amino acid release and alterations in ionic homeostasis contribute to both necrotic and apoptotic neuronal death. Apoptosis is distinguished from necrosis as gene activation is the predominant mechanism regulating cell survival. Following hypoxic-ischemic episodes in the brain, genes that promote as well as inhibit apoptosis are activated. It is the balance in the expression of pro- and anti-apoptotic genes that likely determines the fate of neurons exposed to hypoxia. The balance in expression of pro- and anti-apoptotic genes may also account for the regional differences in vulnerability to hypoxic insults [11]. The gray matter is highly susceptible to acute hypoxic-ischemic injury given the predominance of neuronal cell bodies in this region. The white matter, however, is somewhat resilient to the effects of acute hypoxia which explains the characteristic delayed course of DPHL [12]. DPHL is also believed to occur after the episode of mild to moderate hypoxia, in contrast to severe hypoxia which typically results in acute hypoxic-ischemic injury affecting gray matter [1]. Severe hypoxic-ischemic injury most commonly affects the areas of basal ganglia, thalami, neocortex, and hippocampus producing characteristic pattern of abnormalities on MRI [13]. In contrast, DPHL seems to be observed radiographically in the white matter zones. DPHL has been reported in the setting of decreased levels of arylsulfatase A, thus suggesting a possible enzymatic mechanism [14].

Another proposed explanation relates to the turn-over rates of myelin-related proteins which usually range from 19 to 22 days that correlates well with the timeline of occurrence of neurological deficits [15]. Myelin-related proteins were elevated in both of our patients, and the occurrence of neurological symptoms was in concordance with the reported time range of protein turnover. This may have been the underlying phenomenon for DPHL development in our patients. MRI findings in DPHL shows a wide range of white matter abnormalities such as watershed infarcts, widespread ischemic changes and diffuse hyperintensities in cerebral white matter predominantly in the region of dorsal frontal and parietal lobe bilaterally. CSF analysis can be a valuable diagnostic modality in certain patients showing elevated myelin basic protein, a marker of acute widespread demyelination from the hypoxic region [2]. The majority of patients who survive the initial hospitalization period demonstrate significant recovery, although a significant number of patients have some cognitive or other neurologic deficits [4, 5]. Lasting deficits in frontal-executive functions such as attention and a declined mental flexibility are most common symptoms. Supportive care is the mainstay of treatment during the period of neurological decline.

## Conclusions

DPHL is a unique clinical entity with characteristic clinical and imaging findings. Diagnosis requires a high degree of clinical suspicion. The clinical presence of delayed onset encephalopathy following an acute hypoxic insult together with MRI brain imaging showing white matter disease and elevated CSF myelin basic protein are collectively suggestive of DPHL. Majority patients demonstrate complete recovery, however, lasting neurological deficits are known to occur with DPHL.
